# Differences in psychometric characteristics of outpatients with somatic symptom disorder from general hospital biomedical (neurology/gastroenterology), traditional Chinese medicine, and psychosomatic settings

**DOI:** 10.3389/fpsyt.2023.1205824

**Published:** 2023-07-19

**Authors:** Dandan Ma, Wei Lu, Kurt Fritzsche, Anne Christin Toussaint, Tao Li, Lan Zhang, Yaoyin Zhang, Hua Chen, Heng Wu, Xiquan Ma, Wentian Li, Jie Ren, Rainer Leonhart, Jinya Cao, Jing Wei

**Affiliations:** ^1^Department of Psychological Medicine, Peking Union Medical College Hospital, Chinese Academy of Medical Sciences and Peking Union Medical College, Beijing, China; ^2^Department of Psychosomatic Medicine, Beijing Hospital of Traditional Chinese Medicine, Capital University, Beijing, China; ^3^Center for Mental Health, Department of Psychosomatic Medicine and Psychotherapy, Faculty of Medicine, Medical Centre – University of Freiburg, Freiburg, Germany; ^4^Department of Psychosomatic Medicine and Psychotherapy, University Medical Centre Hamburg-Eppendorf, Hamburg, Germany; ^5^Mental Health Centre, West China Hospital, Sichuan University, Chengdu, China; ^6^Department of Psychosomatic Medicine, Sichuan Provincial People’s Hospital, University of Electronic Science and Technology of China, Chengdu, China; ^7^Department of Psychological Medicine, Zhong Shan Hospital, Fudan University, Shanghai, China; ^8^Department of Psychosomatic Medicine, Tongji Hospital, School of Medicine, Tongji University, Shanghai, China; ^9^Department of Psychosomatic Medicine, Dongfang Hospital, School of Medicine, Tongji University, Shanghai, China; ^10^Department of Clinic Psychology, Wuhan Mental Health Centre, Wuhan, China; ^11^Department of Rehabilitation, General Hospital of Jincheng Anthracite Coal Mining Group Co. Ltd., Jincheng, China; ^12^Institute of Psychology, University of Freiburg, Freiburg, Germany

**Keywords:** somatic symptom disorder, psychometric, biomedical, traditional Chinese medicine, psychosomatic

## Abstract

**Objective:**

The aim of this study is to investigate the psychometric characteristics of outpatients diagnosed with somatic symptom disorder (SSD) in biomedical, Traditional Chinese Medicine (TCM) and psychosomatic settings.

**Materials and methods:**

A total of 697 participants who completed SCID-5 and questionnaires were presented in our former study, as 3 of them had missed questionnaire data, a total of 694 participants are presented in this study. A secondary analysis of the psychometric characteristics of Somatic Symptom Disorder–B Criteria Scale (SSD-12), Somatic Symptom Severity Scale of the Patient-Health Questionnaire (PHQ-15), Patient Health Questionnaire-9 (PHQ-9) and General Anxiety Disorder-7 (GAD-7) is done to compare differences among outpatients from the three settings of medical specialties.

**Results:**

Based on the DSM-5 criteria, 90 out of 224 (40.2%) participants enrolled in biomedical departments (represented by neurology and gastroenterology departments), 44/231 (19.0%) in TCM departments, and 101/239 (42.3%) in the psychosomatic medicine departments were diagnosed with SSD. The scores of PHQ-15 in the biomedical, TCM and psychosomatic settings were 11.08 (± 4.54), 11.02 (± 5.27) and 13.26 (± 6.20); PHQ-9 were 10.43 (± 6.42), 11.20 (± 5.46) and 13.42 (± 7.32); GAD-7 were 8.52 (± 6.22), 9.57 (± 5.06) and 10.83 (± 6.24); SSD-12 were 22.26 (± 11.53), 22.98 (± 10.96) and 25.03 (± 11.54) respectively. The scores of PHQ-15, PHQ-9 and GAD-7 in SSD patients were significantly higher in psychosomatic departments than that in biomedical settings (*p* < 0.05). The cutoff point for SSD-12 was ≥16 in total patients; 16, 16, 17 in biomedical, TCM and psychosomatic settings, respectively. The cutoff point for PHQ-15 was found to be ≥8 in total patients; 8, 9, 11 in biomedical, TCM and psychosomatic settings, respectively.

**Conclusion:**

SSD patients from psychosomatic departments had higher level of somatic symptom severity, depression and anxiety than from TCM and biomedical settings. In our specific sample, a cutoff point of ≥16 for SSD-12 could be recommended in all three settings. But the cutoff point of PHQ-15 differs much between different settings, which was ≥8, 9, and 11 in biomedical, TCM, and psychosomatic settings, respectively.

## 1. Introduction

In DSM-5, somatic symptom disorder (SSD) was introduced in a new section, “somatic symptoms and related disorders,” replacing the category of “somatoform disorders” in DSM-IV (1, 2). SSD is defined as one or more persistent physical symptoms that cause distress to patients or significantly disrupt their daily life. This new category eliminated the requirement that somatic symptoms must be “medically unexplained.” The focus shifted to the psychological responses to somatic symptoms (3). Research has shown that SSD is not only common in psychiatric settings, but also in other biomedical settings, especially in neurology (4, 5), gastroenterology (6), cardiology (7) and otorhinolaryngology (8) clinics. The prevalence rates of SSD are reported as from 5 to 93.1% (9, 10). Patients usually show high levels of medical resources usage, repeated visits to hospitals, excessive tests and treatment, difficult doctor-patient relationship, social functional impairment and high socio-economic cost (10). Therefore, screening and recognition of SSD in different clinical settings is of great importance.

At present, little is known about the difference of SSD in different clinical settings. Our former study showed that the prevalence of SSD was 40.2, 19.0 and 42.1% in the modern biomedical settings, Traditional Chinese Medicine (TCM) departments and psychological medicine departments, respectively (11). The prevalence rates of SSD may be higher in patients with functional disabilities, such as fibromyalgia syndrome, functional gastrointestinal disease, and chronic fatigue syndrome (12). A study comparing the clinical features of patients with SSD attending psychiatric services and rheumatology outpatient services found that rheumatology outpatients had increased somatosensory amplification, hypochondria, amenorrhea, higher stigmatization attitudes toward mental illness, poorer quality of life and higher degree of disability (13). This suggests different psychometric characteristics in different patient groups.

The Structured Clinical Interview for DSM-5 (SCID-5) can be applied as the reference standard for SSD diagnosis (14). But it takes a relatively long time to complete and therefore is not commonly recommended in busy outpatient settings. Some scales have shown good efficiency for SSD screening, especially SSD-12 and PHQ-15 (15). SSD-12 was developed to assess the psychological B criteria of SSD and has been to verified in community and clinical samples among several countries. Studies showed different cutoff point varied from 14 to 26 (16, 17), depending on different clinical settings. PHQ-15 is a questionnaire assessing SSD Criterion A, which identify patients with elevated symptom burden (18). Studies have found that total score and cutoff point of PHQ-15 in determined SSD patients also varied in different clinical settings (19, 20).

SSD is usually accompanied with other psychiatric symptoms, especially depression and/or anxiety. Studies investigating the level of depression and/or anxiety in patients diagnosed with SSD found that SSD patients tend to have higher scores of the PHQ-9 and/or GAD-7 (21). Prevalence of depression decreased from psychosomatic, biomedical to TMC settings, and the scores of PHQ-9 also showed a corresponding trend (22). Studies have found that the severity of somatic symptoms was closely related to anxiety (23, 24). But only limited information could be found about the differences of these clinical variables between psychiatric departments and the other clinical settings.

Thus, the aims of the present study are: (1) to compare psychometric characteristics of SSD-12, PHQ-15, PHQ-9 and GAD-7 in patients diagnosed with SSD in the biomedical, TMC and psychosomatic departments; (2) to identify the cutoff point of SSD-12 and PHQ-15 for the diagnose of SSD among the three different clinical settings.

## 2. Materials and methods

### 2.1. Study design and settings

Our former multicenter cross-sectional study was performed during May 2016 to March 2017 in the outpatients of nine tertiary hospitals in North, North-Central, East, Central, and West China (Beijing, Jincheng, Shanghai, Wuhan, and Chengdu, respectively). The study included three clinical settings, and approximately equal numbers of participants were recruited. The neurology and gastroenterology departments represented the modern biomedical settings, the TCM departments represented the traditional medical settings, and the psychological medicine departments represented the psychosomatic medical settings. The study design was approved by the Ethics Committees of Peking Union Medical College Hospital and the University Medical Centre Freiburg, Germany.

### 2.2. Subjects

The participants were consecutively enrolled in each center until similar numbers of patients are recruited in the three different settings. All participants recruited in the study were informed of the details of the study through an information booklet. The participants were fully informed that their data would be analyzed anonymously. All participants signed an informed consent. The inclusion criterion were adults at least 18 years old, visiting for treatment voluntarily, being able to read and have adequate writing skills to sign the informed consent form. The exclusion criteria were as follows: language barriers, limited writing skills, cognitive impairment, acute psychosis or suicidal tendency.

All patients were interviewed use the diagnostic SCID-5 by trained clinical researchers to assess whether they with SSD or not.

Detailed information about the project process can be found in our previous study (11).

### 2.3. Instruments

The following scales were used to assess the psychometric characteristics of patients with SSD.

#### 2.3.1. Somatic symptom disorder–B criteria scale

The Somatic symptom disorder–B criteria scale (SSD-12) is a 12-item questionnaire which developed as a direct measure of the B criteria of SSD (23). It is a five-point Likert scale, each item rated from 0 (“not at all”) to 4 (“very often”). The total score is between 0 and 48 points. The Chinese version of the SSD-12 used in present study has been validated in previous research with a Cronbach’s alpha of 0.95 (25).

#### 2.3.2. Somatic symptom severity scale of the patient – health questionnaire (PHQ-15)

The PHQ-15 is a self-report questionnaire that measures the A-criteria of SSD. The PHQ-15 consists of 15 somatic symptoms and assesses the distress of each symptom in the prior four weeks (18). Each item rated from 0 (“not bothered at all”) to 2 (“bothered a lot”), resulting in a total score ranging from 0 to 30. Both Western and Chinese versions of PHQ-15 have been verified to have good reliability and validity (11, 19).

#### 2.3.3. Patient-health-questionnaire-9

The atient-health-questionnaire-9 (PHQ-9) is a self-report questionnaire used to assess the severity of depressive symptoms in the last two weeks (26). The PHQ-9 consists of nine items, each item is scored from 0 (not at all) to 3 (nearly every day), resulting in a total score ranging from 0 to 27. Higher score represents more severe depressive symptoms. The Chinese version of PHQ-9 has been shown to be a reliable and valid instrument in general health care (22).

#### 2.3.4. General anxiety disorder-7

The General anxiety disorder-7 (GAD-7) is a 7-item questionnaire which used to assess the presence of signs and symptoms of anxiety disorder and related symptoms (24). Each item is scored from 0 (not at all) to 3 (nearly every day). The total score of GAD-7 is 0 to 21, and the higher score represents more severe anxiety symptoms. The GAD-7 showed good reliability and validity in Chinese patients (27).

### 2.4. Statistical procedures

Descriptive data are presented as the means and standardized deviations for continuous variables and percentages for categorical variables. For normally distributed continuous variables, the independent samples *t*-test were used to test the difference between two groups, and one-way analysis of variance (ANOVA) were adopted to test the difference between three independent groups. The Bonferroni method was used for multiple comparisons. The Chi-squared test or fisher’s precise test was used for categorical variables. A value of p of less than 0.05 (two-tailed) was considered significant. Pearson correlation coefficient was used to test the correlation among clinical variables, SSD-12, PHQ-15, PHQ-9, and GAD-7. We explored the potential cutoff points and plotted receiver operating characteristic (ROC) curves for each of these scales. The highest Youden Index was calculated and used to establish the best cut-off.

All statistical analyses were performed with IBM SPSS Statistics 25.0.

## 3. Results

### 3.1. Demographic characteristics

A total of 697 participants who completed SCID-5 and questionnaires were presented in our former study, as 3 of them had missed questionnaire data, a total of 694 participants are presented in this study. Among them, 90 out of 224 participants in biomedical settings, 44 out of 231 participants in TCM departments, and 101 out of 239 participants in psychosomatic departments were diagnosed with SSD. The prevalence of SSD was 40.2, 19.0 and 42.3% in the biomedical, TCM and psychosomatic settings, respectively. The differences were significant (χ^2^ = 34.153, *p* ≤ 0.001).

The differences were significant in terms of age and occupation between participants diagnosed with SSD in modern biomedical settings and psychosomatic medicine departments. No significant differences were observed in terms of other demographic characteristics among participants diagnosed with SSD in these three different settings.

There were no significant differences in age, gender, health insurance, residence, marital status, family income, occupation, education, smoking history, alcohol consumption and exercise habits between patients with and without SSD in the modern biomedical settings, TCM departments and psychosomatic medicine departments, respectively (Table 1).

**Table 1:** Sociodemographic and lifestyle data.

	Bio			TCM			Psycho			*P_1_*	*P_2_*	*P_3_*
	SSD (*n* = 90)	Non-SSD (*n* = 134)	*P*	SSD (*n* = 44)	Non-SSD (*n* = 187)	*P*	SSD (*n* = 101)	Non-SSD (*n* = 138)	*P*			
Age (M ± SD)	47.1 ± 14.8	45.5 ± 14.6	0.439	42.3 ± 12.1	43.9 ± 13.5	0.480	39.6 ± 13.3	38.9 ± 14.3	0.699	0.001^*^	0.185	0.785
Female (%)	53.3	57.5	0.542	70.5	68.4	0.796	62.4	57.2	0.425	0.618	0.174	1.000
Health insurance (yes, %)	88.9	91.8	0.465	86.4	86.1	0.963	81.8	85.7	0.423	0.516	1.000	1.000
Residence (%)			0.063			0.898			0.469	1.000	0.639	0.189
City	80.0	81.3		88.6	89.3		75.0	79.0				
Country	20.0	18.7		11.4	10.7		25.0	21.0				
Marital status (%)			0.467			0.699				0.504	0.987	0.798
Single	15.6	12.7		15.9	13.4		23.8	30.4				
Married	73.3	81.3		79.5	80.2		60.4	59.4				
Separated	0	0		2.3	0.5		2.0	0				
Divorced	6.7	3.7		2.3	4.8		9.9	5.1				
Widowed	4.4	2.2		0	0.5		2.0	1.4				
Others	0	0		0	0.5		2.0	3.6				
Family income (%)			0.587			0.291			0.207	0.630	1.000	1.000
Low (under 4,000 RMB)	35.6	31.3		38.6	27.3		40.0	37.5				
Middle (under 4,000 RMB)	30.0	36.6		36.4	39.0		37.0	29.4				
High (above 8,000 RMB)	34.4	32.1		25.0	33.7		23.0	33.1				
Occupation (%)			0.171			0.168			0.800	0.001^*^	1.000	0.258
Employed	46.7	48.5		43.2	58.8		43.6	44.2				
Unemployed	13.3	7.5		15.9	7.0		18.8	16.7				
Retire	32.2	25.4		25.0	23.5		10.9	14.5				
Housewife	3.3	8.2		4.5	4.3		10.9	6.5				
Student	2.2	3.0		2.3	2.7		10.9	11.6				
Others	2.2	7.5		9.1	3.7		5.0	6.5				
Education (%)			0.322			0.095			0.806	1.000	0.963	1.000
Primary school	6.7	11.2		6.8	1.6		8.9	6.5				
Middle school	28.9	20.1		18.2	12.8		19.8	21.7				
Higher school	22.2	20.1		36.4	31.6		21.8	25.4				
University or higher	42.2	48.5		38.6	54.0		49.5	46.4				
Smoking history (%)			0.939			0.080			0.268	1.000	1.000	1.000
Never	67.8	69.4		65.9	81.3		74.0	68.8				
In the past	15.6	15.7		15.9	8.0		10.0	17.4				
Currently	16.7	14.9		18.2	10.7		16.0	13.8				
Alcohol consumption (%)			0.800			0.131			0.474	1.000	0.777	1.000
Never	51.1	44.8		56.8	50.3		56.0	46.4				
Social drinking	42.2	46.3		29.5	43.3		37.0	44.9				
Drink in the past, but quit now	3.3	4.5		11.4	4.3		5.0	7.2				
Almost drink everyday	3.3	4.5		2.3	2.1		2.0	1.4				
Physical activities in winter (%)			0.355			0.359			0.714	1.000	1.000	0.864
Regular exercise, > 2 h	20.0	29.1		25.0	20.9		17.8	14.5				
Regular exercise, 1–2 h	14.4	15.7		15.9	18.2		11.9	16.7				
Regular exercise, < 2 h	36.7	27.6		25.0	36.9		41.6	39.9				
no exercise	28.9	27.6		34.1	24.1		28.7	29.0				
Physical activities in summer (%)			0.412			0.913			0.919	1.000	1.000	1.000
Regular exercise, > 2 h	24.4	32.8		31.8	27.8		18.8	22.5				
Regular exercise, 1–2 h	32.2	23.9		20.5	25.1		25.7	25.4				
Regular exercise, < 2 h	21.1	23.1		29.5	29.4		29.7	27.5				
No exercise	22.2	20.1		18.2	17.6		25.7	24.6				

Bio, Biomedical Settings; TCM, Traditional Chinese Medicine Settings; Psycho, Psychosomatic Settings; SSD, somatic symptom disorders. Italic values indicate significance of *p* value (*p* < 0.05). Post hoc analysis was adjusted by Bonferroni. *P*_1_, Biomedical Settings vs. Psychosomatic Settings. *P*_2_, Biomedical Settings vs. Traditional Chinese medicine Settings. *P*_3_, Traditional Chinese medicine Settings vs. Psychosomatic Settings. *p* < 0.05^*^.

### 3.2. Psychometric characteristics

The scores of PHQ-15 were 11.08 (± 4.54), 11.02 (± 5.27) and 13.26 (± 6.20) in the biomedical, TCM and psychosomatic settings, respectively. The scores of PHQ-9 were 10.43 (± 6.42), 11.20 (± 5.46) and 13.42 (± 7.32) respectively. The scores of GAD-7 were 8.52 (± 6.22), 9.57 (± 5.06) and 10.83 (± 6.24) respectively. The scores of SSD-12 were 22.26 (± 11.53), 22.98 (± 10.96), 25.03 (± 11.54), respectively. Significant differences in scores of PHQ-15, PHQ-9 and GAD-7 were identified between patients diagnosed with SSD in the biomedical, TCM and psychosomatic settings (*p* < 0.05). The scores of PHQ-15, PHQ-9 and GAD-7 were significantly higher among SSD patients in psychosomatic departments than that in biomedical settings (*p* < 0.05). However, no difference was found in SSD-12 scores among patients with SSD in the biomedical, TCM and psychosomatic settings (Table 2).

**Table 2:** Relationship between clinical variables and settings in patients diagnosed with SSD.

	Bio	TCM	Psycho	*p_1_*	*p_2_*	*p_3_*
SSD–B Criteria SSD-12	22.26 ± 11.53	22.98 ± 10.96	25.03 ± 11.54	0.286	1.000	0.964
Somatic symptoms severity PHQ-15	11.08 ± 4.54	11.02 ± 5.27	13.26 ± 6.20	0.018^*^	1.000	0.070
Depression PHQ-9	10.43 ± 6.42	11.20 ± 5.46	13.42 ± 7.32	0.007^*^	1.000	0.204
Anxiety GAD-7	8.52 ± 6.22	9.57 ± 5.06	10.83 ± 6.24	0.027^*^	1.000	0.746

Bio, Biomedical Settings; TCM, Traditional Chinese medicine Settings; Psycho, Psychosomatic Settings; SSD-12, Somatic Symptom Disorder B criteria; PHQ-15, Patient Health Questionnaire15; PHQ-9, Patient Health Questionnaire-9; GAD-7, General Anxiety Disorder-7. The total score of SSD-12 is between 0 and 48 points, and higher than 16 indicates SSD risk in Chinese version. The total score of PHQ-15 ranges from 0 to 30 and represents the severity of somatic symptoms, ranging from minimal (0 to 4), mild (5 to 9), moderate (10 to 14) to severe (15 to 30). A higher score represents higher symptom burdens for all scales. Italic values indicate significance of *p* value (*p* < 0.05). Post hoc analysis was adjusted by Bonferroni. P_1_, Biomedical Settings vs. Psychosomatic Settings; P_2_, Biomedical Settings vs. Traditional Chinese medicine Settings; P_3_, Traditional Chinese medicine Settings vs. Psychosomatic Settings. *p* < 0.05^*^.

There were significant differences in scores of SSD-12, PHQ-15, PHQ-9, and GAD-7 between patients with and without SSD in the modern biomedical settings, TCM departments and psychosomatic medicine departments, respectively (Supplementary Tables S0–S2).

### 3.3. Correlation between clinical variables

Pearson correlation analysis showed that PHQ-9 and GAD-7 were highly correlated. The SSD-12 score, PHQ-15 score were moderately correlated with the scores of PHQ-9 and GAD-7 (Supplementary Table S3).

### 3.4. Symptom profile of the participants as assessed by using PHQ-15

The physical symptom profile of SSD patients in different settings were analyzed and the result showed that patients in the TCM departments reported significantly more menstrual problems than patients in biomedical settings, and headaches, chest pain and pain or problems during sexual intercourse were more common in psychosomatic settings than in biomedical settings. No significant differences were observed in terms of other symptoms (Supplementary Table S4).

### 3.5. The cutoff points of SSD-12 and PHQ-15 for the diagnoses of SSD in the three settings

**Figure 1:**
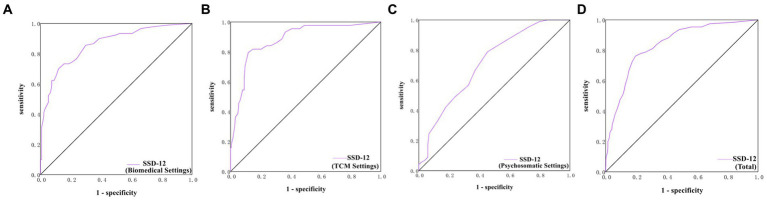
ROC curves of SSD-12 in the biomedical, TCM and psychosomatic settings. **(A)** ROC curve of SSD-12 in the biomedical settings. **(B)** ROC curve of SSD-12 in the TCM settings. **(C)** ROC curve of SSD-12 in the psychosomatic settings. **(D)** ROC curve of SSD-12 in the total settings.

The cutoff point with the highest Youden-Index for SSD-12 was found to be ≥16 in total patients, with an area under the curve (AUC) of 0.84, sensitivity of 76.17%, specificity of 80.39%, and Youden Index of 0.57; and the cutoff point for SSD-12 were found to be 16, 16, 17 in biomedical, TCM and psychosomatic settings, respectively (Table 3; Supplementary Table S5A; Figures 1A–D).

**Table 3:** The cutoff points of SSD-12 for the diagnoses of SSD in the three settings.

SSD-12	Cutoff	AUC (95 %CI)	Youden Index	SE (%) (95 %CI)	SP (%) (95 %CI)	PPV (%) (95 %CI)	NPV (%) (95 %CI)	PTP	*p*
Bio	16	0.86 (0.81, 0.91)	0.58	70.00 (60.35, 79.65)	88.06 (82.50, 93.62)	79.75 (70.69, 88.81)	81.38 (74.97, 87.79)	40.18%	<0.001
TCM	16	0.88 (0.83, 0.94)	0.68	79.55 (67.14, 91.95)	88.24 (83.57, 92.90)	61.40 (48.37, 74.44)	94.83 (91.50, 98.15)	19.05%	<0.001
Psycho	17	0.76 (0.70, 0.82)	0.44	78.22 (70.03, 86.41)	65.94 (57.94, 73.95)	62.70 (54.14, 71.26)	80.53 (73.12, 87.94)	42.26%	<0.001
Total	16	0.84 (0.81, 0.87)	0.57	76.17 (70.68, 81.66)	80.39 (76.75, 84.04)	66.54 (60.87, 72.22)	86.82 (83.59, 90.05)	33.86%	<0.001

SSD-12, Somatic Symptom Disorder B criteria; Bio, Biomedical Settings; TCM, Traditional Chinese medicine Settings; Psycho, Psychosomatic Settings; Total, Patients from all three settings; Cutoff, The best cutoff point, which was established by calculating the highest Youden Index; AUC, Area Under the Curve; SE, Sensitivity; SP, Specificity; PPV, Positive Predictive Values; NPV, Negative Predictive Values; CI, confidence intervals; PTP, Pre-test probability. Italic values indicate significance of *p* value (*p* < 0.001).

**Figure 2:**
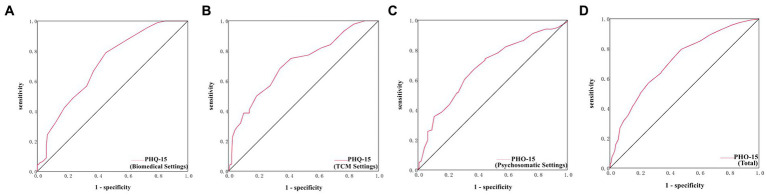
ROC curves of PHQ-15 in the biomedical, TCM and psychosomatic settings. **(A)** ROC curve of PHQ-15 in the biomedical settings. **(B)** ROC curve of PHQ-15 in the TCM settings. **(C)** ROC curve of PHQ-15 in the psychosomatic settings. **(D)** ROC curve of PHQ-15 in the total settings.

The cutoff point with the highest Youden-Index for PHQ-15 was found to be ≥8 in total patients, with an AUC of 0.72, sensitivity of 79.57%, specificity of 52.07%, and Youden Index of 0.32; and the cutoff point for PHQ-15 were found to be 8, 9, 11 in biomedical, TCM and psychosomatic settings, respectively (Table 4; Supplementary Table S5B; Figures 2A–D).

**Table 4:** The cutoff points of PHQ-15 for the diagnoses of SSD in the three settings.

PHQ-15	Cutoff	AUC (95 %CI)	Youden Index	SE (%) (95 %CI)	SP (%) (95 %CI)	PPV (%) (95 %CI)	NPV (%) (95 %CI)	PTP	*p*
Bio	8	0.71 (0.65, 0.78)	0.33	78.89 (70.29, 87.48)	54.48 (45.94, 63.02)	53.79 (45.17, 62.41)	79.35 (70.92, 87.78)	40.18%	<0.001
TCM	9	0.72 (0.63, 0.80)	0.34	68.18 (53.86, 82.51)	65.78 (58.91, 72.64)	31.92 (22.32, 41.51)	89.78 (84.64, 94.92)	19.05%	<0.001
Psycho	11	0.69 (0.62, 0.75)	0.30	67.33 (58.02, 76.63)	62.32 (54.13, 70.51)	56.67 (47.67, 65.66)	72.27 (64.11, 80.43)	42.26%	<0.001
Total	8	0.72 (0.68, 0.76)	0.32	79.57 (74.38, 84.77)	52.07 (47.48, 56.66)	45.95 (41.08, 50.81)	83.28 (78.93, 87.62)	33.86%	<0.001

PHQ-15, Patient Health Questionnaire15; Bio, Biomedical Settings; TCM, Traditional Chinese medicine Settings; Psycho, Psychosomatic Settings; Total, Patients from all three settings; Cutoff, The best cutoff point, which was established by calculating the highest Youden Index. AUC, Area Under the Curve; SE, Sensitivity; SP, Specificity; PPV, Positive Predictive Values; NPV, Negative Predictive Values; CI, confidence intervals; PTP, Pre-test probability. Italic values indicate significance of *p* value (*p* < 0.001).

## 4. Discussion

Many SSD patients attend non-psychiatric services rather than the psychiatric services (28, 29). Obviously, people choose different medical models based on their disease attribution (30). But are there other differences between these outpatient settings that should be noted by clinicians?

In our study, we recruited patients from three different settings in China. Patients are referred to the biomedical settings mainly for the diagnosis and treatment of somatic symptoms or diseases in the corresponding specialty system. Patients seeking treatment in the settings of psychosomatic medicine are mainly seeking solutions to emotional problems, such as anxiety, depression, or physical symptoms that have not been relieved for a long time accompanied by obvious emotional problems. TCM mainly serves as preventive and modulating medicine (“maintaining health”) in China, patients prone to seek help from TCM when they believe that no significant or urgent medical issues behind their symptoms.

Comparisons of sociodemographic features among SSD patients in different settings showed that the mean age was significantly higher for patients attending the modern biomedical settings than those attending the psychosomatic medicine departments. Consistent with previous studies, which found that patients attending the rheumatology outpatient services had a higher mean age than those attending the psychiatry outpatient services (13). Patients with late onset symptoms may tend think their symptoms are physical and thus are reluctant to seek mental health services. The percentage of retired outpatients in the biomedical settings was significantly higher than psychosomatic departments. We speculated that the distribution of age may be responsible for this phenomenon. There were no significant differences observed in terms of the other demographic characteristics. The age difference itself should warrant investigation into the psychometric characteristics of SSD patients from the three different clinical settings.

In the study by Hüsing et al. (31) with patients from a psychosomatic rehabilitation center in Germany, the SSD-12 score of SSD patients were 22.97 (± 10.98) at baseline. The study by Behm et al. (32) was performed on SSD patients from a psychosomatic outpatient clinic, found that the scores of SSD-12 and GAD-7 were 26.04 (± 10.06) and 11.71 (± 5.41). Li et al. (33) performed the study on breast cancer patients in China, observed that in the patients who can meet SSD diagnosis, the average scores of PHQ-15, PHQ-9, GAD-7 were 9.26 (± 3.86), 10.11 (± 4.09), and 9.19 (± 3.77), respectively. The study by Berens et al. (6) was performed on patients with gastrointestinal complaints, the PHQ-9 scores of SSD patients were 13.2 (± 5.1), and the GAD-7 scores were 10.9 (± 4.9). Tian et al. (8) performed the study on outpatients with SSD in otorhinolaryngology clinics, the scores of PHQ-9 and GAD-7 were 10.2 (± 4.1) and 8.8 (± 3.3), respectively. Xiong et al. (22) conducted the study in patients both with and without multiple somatic symptoms in the settings of biomedical, TCM and psychosomatic medicine, found that the scores of PHQ-9 were different among them and no difference was found in the PHQ-15. Studies in different clinical settings have produced different results. There is a lack of studies that simultaneously compared the level of psychometric features of patients diagnosed with SSD in the different settings.

In this study, SSD patients were diagnosed with SCID-5 in multi-centers. Significantly differences were found in psychometric characteristic of SSD patients in different settings. Scores of PHQ-15, PHQ-9 and GAD-7 among SSD patients in psychosomatic settings were significantly higher than in biomedical settings. Clinically, SSD is usually accompanied with depression/anxiety, and the latter can also increase the attention to physical symptoms and raise the somatic symptoms severity in organic disease patients (34, 35).

No difference was found in SSD-12 scores among the three settings. SSD-12 was developed to directly reflect the SSD criteria. We speculate that SSD-12 had a higher level of stability in assessing SSD in different clinical settings. The cutoff point for SSD-12 was found to be ≥16 in total patients, and 16, 16, 17 in the biomedical, TCM and psychosomatic departments, respectively. Other studies performed in the outpatients of general hospitals in China also found the cutoff point was ≥16/17 for the SSD-12 (36). The study conducted by Abasi et al. (16) on community population and patients with SSD or major depressive disorder according to DSM-5, found that the cutoff point greater than 14 was optimal for the SSD-12.

The cutoff point for PHQ-15 was found to be ≥8 in total patients, and 8, 9, 11 in these three different settings, respectively. Liao et al. (37) conducted study on psychiatric outpatients and healthy controls, found the cutoff point was ≥4/5 for the PHQ-15. Toussaint et al. (38) performed the study with psychiatric outpatients, found the optimal combined cutoff points were ≥ 23 for the SSD-12, and ≥ 9 for the PHQ-15. It may partly be due to the different diagnostic instrument used in these studies. But this difference could also be caused by participants selection difference. Thus, different cutoff points may need to be validated in different patient populations.

We found that the specificity of SSD-12 is relatively low for all cut-off values in patients from psychosomatic medicine. And the specificity was 66, 88, 88% at the cutoff point with highest Youden-Index for SSD-12 in psychosomatic, biomedical, TCM settings, respectively. Similar with ours in psychosomatic settings, previous study by Toussaint et al. (38) reported a specificity of 67% at the highest efficiency for SSD-12 detecting SSD in participants recruited from a psychosomatic outpatient clinic. One possible explanation could be that other patients, i.e., patients with depression or anxiety disorder, in psychosomatic settings also scored high in SSD-12, which would decrease the specificity of screening for SSD in this context. Clinically, SSD, depression and anxiety disorder are three distinct diagnostic entities, but some symptomatic overlaps exist. Previous study by Hüsing et al. (39) showed that the total score of SSD-12 was moderately correlated with the score of depression and anxiety on Scale Health-49. In addition, the AUC of SSD-12 is much smaller in the psychosomatic settings. We think this may partly be related to the fact that in our sample of psychosomatic settings, patients also had higher levels of depression and anxiety, which would have influence on somatic discomfort related cognitive-behavioral symptoms. This is consistent with findings from previous studies, in which the AUC score of PHQ-15 was higher when participants meeting the diagnostic criteria for depression were excluded than when not excluded (38, 40). In our study, the negative predictive value was high in the participants from TCM settings, we speculate that the SSD-12 can be used well for a rule-out in the TCM settings.

To sum up, the study shows that the psychometric characteristics of SSD patients are different in biomedical, TCM and psychosomatic settings. SSD-12 is relatively stable for screening in all settings. But different cutoff points should be noted when applying PHQ-15 for SSD screening.

The study had the following limitations: (1) Patients enrolled in this study mainly came from tertiary hospitals in large cities and the inclusion criteria required them had the ability of reading and writing. Thus, most of them lived in urban and had a higher level of education and above average economic income. (2) Our study only selected the neurology and gastroenterology departments to represent biomedical settings, other biomedical departments may show different characteristics. (3) In our study, all participants were Chinese, and the body experience of patients from different culture may be different. In fact, patients from different regions of China have their own description of body experiences. Thus, the results may need to be further confirmed in patients from other countries or cultures.

## 5. Conclusion

In conclusion, this study compared the psychometric characteristics of patients diagnosed with SSD in biomedical, TCM, and psychosomatic settings in China and found that SSD patients in psychosomatic departments had higher level of somatic symptom severity, depression and anxiety. In our specific sample, a cutoff point of ≥16 for SSD-12 could be recommended in all three settings. But the cutoff point of PHQ-15 was found to be ≥8, 9, and 11 in biomedical, TCM, and psychosomatic settings, respectively.

## Data availability statement

The raw data supporting the conclusions of this article will be made available by the authors, without undue reservation.

## Ethics statement

The studies involving human participants were reviewed and approved by Peking Union Medical College Hospital (PUMCH) and the University Medical Center, Freiburg, Germany (Protocol Number: S-K276). The patients/participants provided their written informed consent to participate in this study.

## Author contributions

JW and KF designed this study. JW, TL, LZ, YZ, HW, XM, HC, WLi, and JR coordinated the study. DM drafted the manuscript. DM and WLu contributed to the data analysis, results, and finalized the manuscript. JC and JW made critical reviews and improvement of the draft. All authors contributed to the article and approved the submitted version.

## Funding

This study was funded by the STI2030-Major Projects (2021ZD0202001), the National High Level Hospital Clinical Research Funding (2022-PUMCH-B-093), and the Education Fund for the Reform and Construction of Comprehensive Evaluation and Assessment System in Clinical Medicine (X226105). The supporters had no role in the design, analysis, interpretation, or publication of this study.

## Conflict of interest

JR was employed by General Hospital of Jincheng Anthracite Coal Mining Group Co. Ltd.

The remaining authors declare that the research was conducted in the absence of any commercial or financial relationships that could be construed as a potential conflict of interest.

## Publisher’s note

All claims expressed in this article are solely those of the authors and do not necessarily represent those of their affiliated organizations, or those of the publisher, the editors and the reviewers. Any product that may be evaluated in this article, or claim that may be made by its manufacturer, is not guaranteed or endorsed by the publisher.
