# Supplementary material for: Differences in psychometric characteristics of outpatients with somatic symptom disorder from general hospital biomedical (neurology/gastroenterology), traditional Chinese medicine, and psychosomatic settings
Source: Front Psychiatry. 2023 Jul 19;14:1205824. doi: 10.3389/fpsyt.2023.1205824 (PMC10395119; doi:10.3389/fpsyt.2023.1205824)
Supplement: Supplementary file 1 [file Table_1.DOCX]

Supplementary Tables

**Table S0. Relationship between clinical variables and SSD Diagnosis in the biomedical settings**

|  | **SSD** | **Non-SSD** | ***F*** | ***P*** |
| --- | --- | --- | --- | --- |
| SSD–B Criteria | 22.26 ± 11.53 | 7.25 ± 7.31 | -10.950 | ＜0.001 |
| SSD-12 |  |  |  |  |
| Somatic symptoms severity  PHQ-15 | 11.08 ± 4.54 | 7.60 ± 4.42 | -5.682 | ＜0.001 |
|  |  |  |  |  |
| Depression | 10.43 ± 6.42 | 5.60 ± 4.58 | -6.166 | ＜0.001 |
| PHQ-9 |  |  |  |  |
| Anxiety | 8.52 ± 6.22 | 3.78 ± 4.19 | -6.329 | ＜0.001 |
| GAD-7 |  |  |  |  |

SSD: somatic symptom disorders. SSD-12: Somatic Symptom Disorder B criteria. PHQ-15: Patient Health Questionnaire15. PHQ-9: Patient Health Questionnaire 9. GAD-7: General Anxiety Disorder-7. Italic values indicate significance of p value (*P* < 0.05).

**Table S1 Relationship between clinical variables and SSD Diagnosis in the TCM settings**

|  | **SSD** | **Non-SSD** | ***t*** | ***P*** |
| --- | --- | --- | --- | --- |
| SSD–B Criteria | 22.98 ± 10.96 | 7.16 ± 7.77 | -9.058 | ＜0.001 |
| SSD-12 |  |  |  |  |
| Somatic symptoms severity  PHQ-15 | 11.02 ± 5.27 | 7.06 ± 4.43 | -5.133 | ＜0.001 |
|  |  |  |  |  |
| Depression | 11.20 ± 5.46 | 4.97 ± 4.54 | -7.867 | ＜0.001 |
| PHQ-9 |  |  |  |  |
| Anxiety | 9.57 ± 5.06 | 3.74 ± 4.13 | -7.107 | ＜0.001 |
| GAD-7 |  |  |  |  |

SSD: somatic symptom disorders. SSD-12: Somatic Symptom Disorder B criteria. PHQ-15: Patient Health Questionnaire15. PHQ-9: Patient Health Questionnaire 9. GAD-7: General Anxiety Disorder-7. Italic values indicate significance of p value (*P* < 0.05).

**Table S2. Relationship between clinical variables and SSD Diagnosis in the psychosomatic settings**

|  | **SSD** | **Non-SSD** | ***t*** | ***P*** |
| --- | --- | --- | --- | --- |
| SSD–B Criteria | 25.03 ± 11.54 | 13.42 ± 11.54 | -7.683 | ＜0.001 |
| SSD-12 |  |  |  |  |
| Somatic symptoms severity  PHQ-15 | 13.26 ± 6.20_b_ | 9.55 ± 5.15 | -5.053 | 0.001 |
|  |  |  |  |  |
| Depression | 13.42 ± 7.32_b_ | 10.26 ± 6.75 | -3.435 | 0.001 |
| PHQ-9 |  |  |  |  |
| Anxiety | 10.83 ± 6.24_b_ | 8.15 ± 5.89 | -3.377 | 0.001 |
| GAD-7 |  |  |  |  |

SSD: somatic symptom disorders. SSD-12: Somatic Symptom Disorder B criteria. PHQ-15: Patient Health Questionnaire15. PHQ-9: Patient Health Questionnaire 9. GAD-7: General Anxiety Disorder-7. Italic values indicate significance of p value (*P* < 0.05).

**Table S3. Pearson Correlation Coefficient between clinical variables**

|  | **SSD-12** | **PHQ-15** | **PHQ-9** | **GAD-7** |
| --- | --- | --- | --- | --- |
| SSD-12 | 1 | 0.383^**^ | 0.519^**^ | 0.525^**^ |
| PHQ-15 | 0.383^**^ | 1 | 0.531^**^ | 0.447^**^ |
| PHQ-9 | 0.519^**^ | 0.531^**^ | 1 | 0.758^**^ |
| GAD-7 | 0.525^**^ | 0.447^**^ | 0.758^**^ | 1 |

SSD-12: Somatic Symptom Disorder B criteria. PHQ-15: Patient Health Questionnaire15. PHQ-9: Patient Health Questionnaire 9. GAD-7: General Anxiety Disorder-7. A Pearson Correlation Coefficient of 0.8-1.0 indicates very highly correlated, 0.6-0.8 indicates highly correlated, 0.4-0.6 indicates moderately correlated. *P*＜0.001^**^

**Table S4.** **Symptom profile of the participants as assessed by using PHQ-15**

|  | **Score** | **Bio** | | **TCM** | | **Psycho** | | ***P*** |
| --- | --- | --- | --- | --- | --- | --- | --- | --- |
|  |  | N | % | N | % | N | % |  |
| Stomach pain | 0 | 33 | 36.7 | 17 | 38.6 | 41 | 40.6 | 0.770 |
|  | 1 | 29 | 32.2 | 17 | 38.6 | 36 | 35.6 |  |
|  | 2 | 28 | 31.1 | 10 | 22.7 | 24 | 23.8 |  |
| Back pain | 0 | 46 | 51.1 | 20 | 45.5 | 52 | 51.5 | 0.846 |
|  | 1 | 27 | 30.0 | 15 | 34.1 | 26 | 25.7 |  |
|  | 2 | 17 | 18.9 | 9 | 20.5 | 23 | 22.8 |  |
| Pain in arms, legs, or joints | 0 | 39 | 43.3 | 16 | 36.4 | 43 | 42.6 | 0.919 |
|  | 1 | 33 | 36.7 | 18 | 40.9 | 40 | 39.6 |  |
|  | 2 | 18 | 20.0 | 10 | 22.7 | 18 | 17.8 |  |
| Menstrual problems | 0 | 67_a_ | 79.8 | 23_b_ | 57.5 | 57_b_ | 58.8 | 0.005^*^ |
|  | 1 | 8_a_ | 9.5 | 13_b_ | 32.5 | 21_a,b_ | 21.6 |  |
|  | 2 | 9_a_ | 10.7 | 4_a_ | 10.0 | 19_a_ | 19.6 |  |
| Headaches | 0 | 41_a_ | 45.6 | 22_a_ | 50.0 | 30_a_ | 29.7 | 0.026^*^ |
|  | 1 | 32_a_ | 35.6 | 14_a_ | 31.8 | 35_a_ | 34.7 |  |
|  | 2 | 17_a_ | 18.9 | 8_a,b_ | 18.2 | 36_b_ | 35.6 |  |
| Chest pain | 0 | 48_a_ | 53.3 | 28_a_ | 63.6 | 45_a_ | 44.6 | 0.041^*^ |
|  | 1 | 30_a_ | 33.3 | 11_a_ | 25.0 | 28_a_ | 27.7 |  |
|  | 2 | 12_a_ | 13.3 | 5_a,b_ | 11.4 | 28_b_ | 27.7 |  |
| Dizziness | 0 | 35 | 38.9 | 17 | 38.6 | 38 | 38.0 | 0.492 |
|  | 1 | 40 | 44.4 | 22 | 50.0 | 39 | 39.0 |  |
|  | 2 | 15 | 16.7 | 5 | 11.4 | 23 | 23.0 |  |
| Fainting spells | 0 | 76 | 84.4 | 38 | 86.4 | 77 | 77.0 | 0.523 |
|  | 1 | 11 | 12.2 | 5 | 11.4 | 16 | 16.0 |  |
|  | 2 | 3 | 3.3 | 1 | 2.3 | 7 | 7.0 |  |
| Feeling heart pound or race | 0 | 31 | 34.4 | 16 | 36.4 | 28 | 27.7 | 0.067 |
|  | 1 | 45 | 50.0 | 20 | 45.5 | 40 | 39.6 |  |
|  | 2 | 14 | 15.6 | 8 | 18.2 | 33 | 32.7 |  |
| Shortness of breath | 0 | 44 | 48.9 | 22 | 50.0 | 33 | 32.7 | 0.064 |
|  | 1 | 33 | 36.7 | 17 | 38.6 | 42 | 41.6 |  |
|  | 2 | 13 | 14.4 | 5 | 11.4 | 26 | 25.7 |  |
| sexual intercourse problem | 0 | 74_a_ | 82.2 | 36_a,b_ | 81.8 | 65_b_ | 64.4 | 0.023^*^ |
|  | 1 | 12_a_ | 13.3 | 7_a_ | 15.9 | 23_a_ | 22.8 |  |
|  | 2 | 4_a_ | 4.4 | 1_a_ | 2.3 | 13_a_ | 12.9 |  |
| Constipation, loose bowels, or diarrhea | 0 | 33 | 36.7 | 18 | 40.9 | 37 | 36.6 | 0.858 |
|  | 1 | 39 | 43.3 | 19 | 43.2 | 40 | 39.6 |  |
|  | 2 | 18 | 20.0 | 7 | 15.9 | 24 | 23.8 |  |
| Nausea, gas, or indigestion | 0 | 23 | 25.6 | 13 | 29.5 | 31 | 30.7 | 0.245 |
|  | 1 | 38 | 42.2 | 20 | 45.5 | 30 | 29.7 |  |
|  | 2 | 29 | 32.2 | 11 | 25.0 | 40 | 39.6 |  |
| Feeling tired or having low energy | 0 | 14 | 15.6 | 5 | 11.4 | 10 | 9.9 | 0.360 |
|  | 1 | 34 | 37.8 | 15 | 34.1 | 29 | 28.7 |  |
|  | 2 | 42 | 46.7 | 24 | 54.5 | 62 | 61.4 |  |
| Trouble sleeping | 0 | 22 | 24.4 | 6 | 13.6 | 17 | 16.8 | 0.050 |
|  | 1 | 28 | 31.1 | 18 | 40.9 | 22 | 21.8 |  |
|  | 2 | 40 | 44.4 | 20 | 45.5 | 62 | 61.4 |  |

Bio: Biomedical Settings. TCM: Traditional Chinese Medicine Settings. Psycho: Psychosomatic Settings. The number “0” means “Not bothered at all”; “1” means “Bothered a little”; “2” means “Bothered a lot”. *P* < 0.05^*^.

**Table S5A. Sensitivity, specificity, negative predictive values, positive predictive values of SSD-12 in three different settings**

| **SSD-12 (Bio)** | **SE (%) (95 %CI)** | **SP (%) (95 %CI)** | **PPV (%) (95 %CI)** | **NPV (%) (95 %CI)** |
| --- | --- | --- | --- | --- |
| Score ⩾10 | 85.56 (78.15, 92.96) | 70.15 (62.30, 78.00) | 65.81 (57.09, 74.53) | 87.85 (81.56, 94.14) |
| Score ⩾11 | 81.11 (72.87, 89.36) | 73.13 (65.53, 80.74) | 66.97 (58.00, 75.94) | 85.22 (78.63, 91.80) |
| Score ⩾12 | 76.67 (67.76, 85.57) | 76.12 (68.81, 83.43) | 68.32 (59.09, 77.55) | 82.93 (76.18, 89.67) |
| Score ⩾13 | 74.44 (65.26, 83.63) | 79.10 (72.13, 86.08) | 70.53 (61.19, 79.86) | 82.17 (75.48, 88.86) |
| Score ⩾14 | 73.33 (64.02, 82.65) | 81.34 (74.66, 88.02) | 72.53 (63.18, 81.88) | 81.96 (75.33, 88.58) |
| Score ⩾15 | 73.33 (64.02, 82.65) | 84.33 (78.09, 90.56) | 75.86 (66.69, 85.04) | 82.48 (76.04, 88.93) |
| Score ⩾16 | 70.00 (60.35, 79.65) | 88.06 (82.50, 93.62) | 79.75 (70.69, 88.81) | 81.38 (74.97, 87.79) |
| Score ⩾17 | 65.56 (55.55, 75.56) | 89.55 (84.31, 94.80) | 80.82 (71.57, 90.07) | 79.47 (72.95, 85.99) |
| Score ⩾18 | 62.22 (52.01, 72.43) | 91.05 (86.15, 95.94) | 82.35 (73.06, 91.65) | 78.21 (71.65, 84.76) |
| Score ⩾19 | 62.22 (52.01, 72.43) | 92.54 (88.03, 97.04) | 84.85 (75.97, 93.73) | 78.48 (72.00, 84.96) |
| Score ⩾20 | 58.89 (48.53, 69.25) | 92.54 (88.03, 97.04) | 84.13 (74.85, 93.40) | 77.02 (70.45, 83.59) |
| Score ⩾21 | 54.44 (43.96, 64.93) | 93.28 (88.99, 97.58) | 84.48 (74.88, 94.09) | 75.30 (68.67, 81.93) |
| Score ⩾22 | 52.22 (41.70, 62.74) | 94.78 (90.96, 98.59) | 87.04 (77.78, 96.29) | 74.71 (68.10, 81.31) |
| Score ⩾23 | 51.11 (40.58, 61.64) | 94.78 (90.96, 98.59) | 86.79 (77.37, 96.21) | 74.27 (67.65, 80.89) |
| Score ⩾24 | 47.78 (37.26, 58.30) | 94.78 (90.96, 98.59) | 86.00 (76.04, 95.96) | 72.99 (66.33, 79.65) |
| Score ⩾25 | 43.33 (32.90, 53.77) | 97.02 (94.10, 99.93) | 90.70 (81.65, 99.74) | 71.82 (65.21, 78.44) |
| **SSD-12 (TCM)** | **SE (%) (95 %CI)** | **SP (%) (95 %CI)** | **PPV (%) (95 %CI)** | **NPV (%) (95 %CI)** |
| Score ⩾10 | 86.36 (75.81, 96.92) | 68.98 (62.29, 75.68) | 39.58 (29.62, 49.54) | 95.56 (92.03, 99.08) |
| Score ⩾11 | 84.09 (72.84, 95.34) | 73.26 (66.86, 79.66) | 42.53 (31.93, 53.13) | 95.14 (91.58, 98.69) |
| Score ⩾12 | 84.09 (72.84, 95.34) | 75.94 (69.75, 82.12) | 45.12 (34.12, 56.12) | 95.30 (91.86, 98.74) |
| Score ⩾13 | 81.82 (69.96, 93.68) | 79.68 (73.86, 85.50) | 48.65 (36.99, 60.31) | 94.90 (91.43, 98.38) |
| Score ⩾14 | 81.82 (69.96, 93.68) | 81.82 (76.24, 87.40) | 51.43 (39.43, 63.43) | 95.03 (91.64, 98.42) |
| Score ⩾15 | 81.82 (69.96, 93.68) | 85.03 (79.87, 90.19) | 56.25 (43.76, 68.74) | 95.21 (91.94, 98.48) |
| Score ⩾16 | 79.55 (67.14, 91.95) | 88.24 (83.57, 92.90) | 61.40 (48.37, 74.44) | 94.83 (91.50, 98.15) |
| Score ⩾17 | 72.73 (59.03, 86.42) | 89.84 (85.47, 94.21) | 62.75 (49.01, 76.48) | 93.33 (89.65, 97.01) |
| Score ⩾18 | 70.46 (56.42, 84.49) | 90.37 (86.11, 94.64) | 63.27 (49.27, 77.26) | 92.86 (89.08, 96.63) |
| Score ⩾19 | 61.36 (46.39, 76.34) | 90.91 (86.75, 95.07) | 61.36 (46.39, 76.34) | 90.91 (86.75, 95.07) |
| Score ⩾20 | 54.55 (39.23, 69.86) | 90.91 (86.75, 95.07) | 58.54 (42.79, 74.28) | 89.47 (85.07, 93.88) |
| Score ⩾21 | 54.55 (39.23, 69.86) | 91.98 (88.05, 95.91) | 61.54 (45.56, 77.52) | 89.58 (85.22, 93.94) |
| Score ⩾22 | 52.27 (36.91, 67.63) | 92.51 (88.71, 96.32) | 62.16 (45.77, 78.56) | 89.18 (84.76, 93.59) |
| Score ⩾23 | 50.00 (34.62, 65.38) | 93.05 (89.37, 96.73) | 62.86 (46.02, 79.70) | 88.78 (84.32, 93.23) |
| Score ⩾24 | 47.73 (32.37, 63.09) | 93.58 (90.04, 97.13) | 63.64 (46.31, 80.96) | 88.38 (83.88, 92.89) |
| Score ⩾25 | 45.46 (30.14, 60.77) | 94.65 (91.40, 97.91) | 66.67 (48.76, 84.57) | 88.06 (83.54, 92.58) |
| **SSD-12 (Psycho)** | **SE (%) (95 %CI)** | **SP (%) (95 %CI)** | **PPV (%) (95 %CI)** | **NPV (%) (95 %CI)** |
| Score ⩾10 | 87.13 (80.48, 93.77) | 47.83 (39.39, 56.27) | 55.00 (47.21, 62.79) | 83.54 (75.19, 91.90) |
| Score ⩾11 | 86.14 (79.28, 92.99) | 50.73 (42.28, 59.17) | 56.13 (48.23, 64.03) | 83.33 (75.20, 91.47) |
| Score ⩾12 | 84.16 (76.91, 91.40) | 52.17 (43.73, 60.61) | 56.29 (48.29, 64.29) | 81.82 (73.60, 90.04) |
| Score ⩾13 | 83.17 (75.75, 90.59) | 55.07 (46.67, 63.48) | 57.53 (49.42, 65.65) | 81.72 (73.72, 89.72) |
| Score ⩾14 | 82.18 (74.59, 89.77) | 55.80 (47.41, 64.19) | 57.64 (49.47, 65.81) | 81.05 (73.03, 89.08) |
| Score ⩾15 | 80.20 (72.29, 88.10) | 58.70 (50.38, 67.01) | 58.70 (50.38, 67.01) | 80.20 (72.29, 88.10) |
| Score ⩾16 | 80.20 (72.29, 88.10) | 62.32 (54.13, 70.51) | 60.90 (52.50, 69.30) | 81.13 (73.56, 88.70) |
| Score ⩾17 | 78.22 (70.03, 86.41) | 65.94 (57.94, 73.95) | 62.70 (54.14, 71.26) | 80.53 (73.12, 87.94) |
| Score ⩾18 | 75.25 (66.69, 83.81) | 67.39 (59.47, 75.31) | 62.81 (54.07, 71.55) | 78.81 (71.33, 86.30) |
| Score ⩾19 | 72.28 (63.40, 81.16) | 68.84 (61.02, 76.67) | 62.93 (54.01, 71.85) | 77.24 (69.72, 84.75) |
| Score ⩾20 | 70.30 (61.23, 79.36) | 71.01 (63.35, 78.68) | 63.96 (54.89, 73.04) | 76.56 (69.12, 84.00) |
| Score ⩾21 | 67.33 (58.02, 76.63) | 71.74 (64.13, 79.35) | 63.55 (54.28, 72.82) | 75.00 (67.52, 82.48) |
| Score ⩾22 | 63.37 (53.81, 72.93) | 72.46 (64.92, 80.01) | 62.75 (53.20, 72.29) | 72.99 (65.46, 80.52) |
| Score ⩾23 | 59.41 (49.66, 69.15) | 73.19 (65.70, 80.67) | 61.86 (52.01, 71.70) | 71.13 (63.58, 78.67) |
| Score ⩾24 | 54.46 (44.58, 64.34) | 76.09 (68.88, 83.29) | 62.50 (52.18, 72.82) | 69.54 (62.11, 76.96) |
| Score ⩾25 | 51.49 (41.57, 61.40) | 78.99 (72.10, 85.87) | 64.20 (53.53, 74.86) | 68.99 (61.70, 76.28) |
| **SSD-12 (Total)** | **SE (%) (95 %CI)** | **SP (%) (95 %CI)** | **PPV (%) (95 %CI)** | **NPV (%) (95 %CI)** |
| Score ⩾10 | 86.38 (81.97, 90.80) | 62.96 (58.53, 67.40) | 54.42 (49.35, 59.50) | 90.03 (86.74, 93.33) |
| Score ⩾11 | 83.83 (79.09, 88.57) | 66.45 (62.11, 70.78) | 56.13 (50.91, 61.34) | 88.92 (85.58, 92.26) |
| Score ⩾12 | 81.28 (76.25, 86.30) | 68.85 (64.59, 73.10) | 57.19 (51.85, 62.52) | 87.78 (84.38, 91.18) |
| Score ⩾13 | 79.57 (74.38, 84.77) | 72.11 (68.00, 76.23) | 59.37 (53.91, 64.82) | 87.34 (83.97, 90.70) |
| Score ⩾14 | 78.72 (73.45, 83.99) | 73.86 (69.82, 77.89) | 60.66 (55.14, 66.17) | 87.15 (83.81, 90.49) |
| Score ⩾15 | 77.87 (72.53, 83.22) | 76.91 (73.04, 80.78) | 63.32 (57.73, 68.91) | 87.16 (83.89, 90.43) |
| Score ⩾16 | 76.17 (70.68, 81.66) | 80.39 (76.75, 84.04) | 66.54 (60.87, 72.22) | 86.82 (83.59, 90.05) |
| Score ⩾17 | 72.34 (66.58, 78.10) | 82.57 (79.09, 86.05) | 68.00 (62.18, 73.82) | 85.36 (82.06, 88.66) |
| Score ⩾18 | 69.36 (63.42, 75.30) | 83.66 (80.27, 87.06) | 68.49 (62.54, 74.43) | 84.21 (80.85, 87.57) |
| Score ⩾19 | 66.38 (60.3, 72.47) | 84.75 (81.45, 88.05) | 69.03 (62.95, 75.10) | 83.12 (79.71, 86.53) |
| Score ⩾20 | 62.98 (56.76, 69.20) | 85.40 (82.16, 88.65) | 68.84 (62.60, 75.08) | 81.84 (78.37, 85.30) |
| Score ⩾21 | 60.00 (53.69, 66.31) | 86.28 (83.11, 89.43) | 69.12 (62.72, 75.51) | 80.82 (77.32, 84.31) |
| Score ⩾22 | 57.02 (50.65, 63.40) | 87.15 (84.07, 90.22) | 69.43 (62.87, 75.99) | 79.84 (76.32, 83.37) |
| Score ⩾23 | 54.47 (48.05, 60.88) | 87.58 (84.55, 90.61) | 69.19 (62.47, 75.90) | 78.98 (75.43, 82.53) |
| Score ⩾24 | 50.64 (44.20, 57.08) | 88.67 (85.76, 91.58) | 69.59 (62.63, 76.56) | 77.82 (74.25, 81.39) |
| Score ⩾25 | 47.23 (40.80, 53.66) | 90.63 (87.96, 93.31) | 72.08 (64.91, 79.24) | 77.04 (73.48, 80.60) |

SSD-12: Somatic Symptom Disorder B criteria. Bio: Biomedical Settings. TCM: Traditional Chinese Medicine Settings. Psycho: Psychosomatic Settings. Total: Patients from all three settings. SE: Sensitivity. SP: Specificity. PPV: Positive Predictive Values. NPV: Negative Predictive Values. CI: confidence intervals.

**Table S5B. Sensitivity, specificity, negative predictive values, positive predictive values of PHQ-15 in three different settings**

| **PHQ-15 (Bio)** | **SE (%) (95 %CI)** | **SP (%) (95 %CI)** | **PPV (%) (95 %CI)** | **NPV (%) (95 %CI)** |
| --- | --- | --- | --- | --- |
| Score ⩾5 | 95.56 (91.22, 99.90) | 26.87 (19.26, 34.47) | 46.74 (39.46, 54.02) | 90.00 (80.28, 99.72) |
| Score ⩾6 | 91.11 (85.12, 97.10) | 34.33 (26.18, 42.47) | 48.24 (40.65, 55.82) | 85.19 (75.40, 94.97) |
| Score ⩾7 | 86.67 (79.51, 93.83) | 41.79 (33.33, 50.25) | 50.00 (42.07, 57.93) | 82.35 (73.06, 91.65) |
| Score ⩾8 | 78.89 (70.29, 87.48) | 54.48 (45.94, 63.02) | 53.79 (45.17, 62.41) | 79.35 (70.92, 87.78) |
| Score ⩾9 | 66.67 (56.74, 76.60) | 62.69 (54.39, 70.98) | 54.55 (45.09, 64.00) | 73.68 (65.48, 81.89) |
| Score ⩾10 | 56.67 (46.23, 67.10) | 67.16 (59.11, 75.22) | 53.68 (43.47, 63.90) | 69.77 (61.74, 77.80) |
| Score ⩾11 | 48.89 (38.36, 59.42) | 76.12 (68.81, 83.43) | 57.90 (46.54, 69.25) | 68.92 (61.38, 76.46) |
| Score ⩾12 | 42.22 (31.82, 52.62) | 82.09 (75.51, 88.67) | 61.29 (48.82, 73.76) | 67.90 (60.64, 75.17) |
| Score ⩾13 | 33.33 (23.40, 43.26) | 87.31 (81.61, 93.02) | 63.83 (49.57, 78.09) | 66.10 (59.06, 73.14) |
| Score ⩾14 | 27.78 (18.34, 37.21) | 91.05 (86.15, 95.94) | 67.57 (51.74, 83.39) | 65.24 (58.35, 72.13) |
| Score ⩾15 | 24.44 (15.39, 33.50) | 93.28 (88.99, 97.58) | 70.97 (54.04, 87.89) | 64.77 (57.97, 71.57) |
| Score ⩾16 | 16.67 (8.82, 24.52) | 94.03 (89.97, 98.09) | 65.22 (44.16, 86.28) | 62.69 (55.94, 69.43) |
| Score ⩾17 | 8.89 (2.90, 14.88) | 94.03 (89.97, 98.09) | 50.00 (22.48, 77.52) | 60.58 (53.88, 67.27) |
| Score ⩾18 | 6.67 (1.41, 11.92) | 96.27 (93.02, 99.52) | 54.55 (19.46,89.63) | 60.56 (53.95, 67.18) |
| Score ⩾19 | 5.56 (0.73, 10.38) | 98.51 (96.43, 100.59) | 71.43 (26.30, 116.56) | 60.83 (54.28, 67.38) |
| Score ⩾20 | 4.44 (0.10, 8.78) | 99.25 (97.78, 100.73) | 80.00 (24.47, 135.53) | 60.73 (54.21, 67.25) |
| **PHQ-15 (TCM)** | **SE (%) (95 %CI)** | **SP (%) (95 %CI)** | **NPV (%) (95 %CI)** | **PPV (%) (95 %CI)** |
| Score ⩾5 | 84.09 (72.84, 95.34) | 32.09 (25.33, 38.84) | 22.56 (16.10, 29.03) | 89.55 (82.03, 97.07) |
| Score ⩾6 | 81.82 (69.96, 93.68) | 38.50 (31.46, 45.54) | 23.84 (16.97, 30.72) | 90.00 (83.28, 96.72) |
| Score ⩾7 | 77.27 (64.38, 90.16) | 46.52 (39.31, 53.74) | 25.37 (17.91, 32.84) | 89.69 (83.53, 95.85) |
| Score ⩾8 | 75.00 (61.68,88.32) | 58.29 (51.16, 65.42) | 29.73 (21.09, 38.37) | 90.83 (85.60, 96.07) |
| Score ⩾9 | 68.18 (53.86, 82.51) | 65.78 (58.91, 72.64) | 31.92 (22.32, 41.51) | 89.78 (84.64, 94.92) |
| Score ⩾10 | 56.82 (41.58, 72.05) | 72.19 (65.71, 78.67) | 32.47 (21.77, 43.17) | 87.66 (82.41, 92.91) |
| Score ⩾11 | 50.00 (34.62, 65.38) | 81.28 (75.64, 86.93) | 38.60 (25.56, 51.63) | 87.36 (82.37, 92.34) |
| Score ⩾12 | 38.64 (23.66, 53.61) | 86.10 (81.09, 91.10) | 39.54 (24.31, 54.76) | 85.64 (80.58, 90.70) |
| Score ⩾13 | 38.64 (23.66, 53.61) | 89.84 (85.47, 94.21) | 47.22 (30.09, 64.35) | 86.15 (81.26, 91.04) |
| Score ⩾14 | 31.82 (17.49, 46.14) | 91.98 (88.05, 95.91) | 48.28 (28.93, 67.62) | 85.15 (80.20, 90.09) |
| Score ⩾15 | 29.55 (15.51, 43.58) | 94.12 (90.71, 97.52) | 54.17 (32.67, 75.66) | 85.02 (80.12, 89.93) |
| Score ⩾16 | 27.27 (13.58, 40.97) | 95.72 (92.79, 98.65) | 60.00 (36.48, 83.52) | 84.83 (79.95, 89.71) |
| Score ⩾17 | 22.73 (9.84, 35.62) | 97.33 (94.99, 99.66) | 66.67 (39.64, 93.69) | 84.26 (79.36, 89.15) |
| Score ⩾18 | 11.36 (1.60, 21.12) | 97.86 (95.77, 99.95) | 55.56 (15.04, 96.07) | 82.43 (77.39, 87.48) |
| Score ⩾19 | 4.55 (-1.86, 10.95) | 97.86 (95.77, 99.95) | 33.33 (-20.86, 87.53) | 81.33 (76.20, 86.46) |
| Score ⩾20 | 4.55 (-1.86, 10.95) | 98.40 (96.58, 100.21) | 40.00 (-28.01, 108.01) | 81.42 (76.31, 86.53) |
| **PHQ-15 (Psycho)** | **SE (%) (95 %CI)** | **SP (%) (95 %CI)** | **NPV (%) (95 %CI)** | **PPV (%) (95 %CI)** |
| Score ⩾5 | 94.06 (89.37, 98.75) | 14.49 (8.55, 20.44) | 44.60 (37.87, 51.33) | 76.92 (59.57, 94.28) |
| Score ⩾6 | 91.09 (85.44, 96.74) | 23.19 (16.06, 30.32) | 46.47 (39.46,53.47) | 78.05 (64.82, 91.28) |
| Score ⩾7 | 86.14 (79.28, 92.99) | 30.44 (22.66, 38.21) | 47.54 (40.24, 54.84) | 75.00 (63.30, 86.70) |
| Score ⩾8 | 82.18 (74.59, 89.77) | 41.30 (32.99, 49.62) | 50.61 (42.88, 58.34) | 76.00 (66.11, 85.89) |
| Score ⩾9 | 78.22 (70.03, 86.41) | 46.38 (37.95, 54.80) | 51.63 (43.63, 59.64) | 74.42 (65.01, 83.83) |
| Score ⩾10 | 73.27 (64.49, 82.05) | 55.07 (46.67, 63.48) | 54.41 (45.93, 62.89) | 73.79 (65.15, 82.42) |
| Score ⩾11 | 67.33 (58.02, 76.63) | 62.32 (54.13, 70.51) | 56.67 (47.67, 65.66) | 72.27 (64.11, 80.43) |
| Score ⩾12 | 60.40 (50.69, 70.10) | 68.84 (61.02, 76.67) | 58.65 (49.03, 68.28) | 70.37 (62.57, 78.17) |
| Score ⩾13 | 51.49 (41.57, 61.40) | 73.91 (66.49, 81.33) | 59.09 (48.61, 69.57) | 67.55 (60.00, 75.10) |
| Score ⩾14 | 43.56 (33.73, 53.40) | 78.99 (72.10, 85.87) | 60.27 (48.78, 71.77) | 65.66 (58.36, 72.96) |
| Score ⩾15 | 38.61 (28.95, 48.27) | 84.06 (77.87, 90.24) | 63.93 (51.53, 76.33) | 65.17 (58.10, 72.24) |
| Score ⩾16 | 35.64 (26.14, 45.15) | 89.13 (83.87, 94.39) | 70.59 (57.65, 83.53) | 65.43 (58.56, 72.29) |
| Score ⩾17 | 26.73 (17.95, 35.51) | 90.58 (85.64, 95.51) | 67.50 (52.33, 82.67) | 62.81 (56.04, 69.59) |
| Score ⩾18 | 25.74 (17.07, 34.42) | 93.48 (89.31, 97.65) | 74.29 (59.05, 89.52) | 63.24 (56.56, 69.91) |
| Score ⩾19 | 19.80 (11.90, 27.71) | 93.48 (89.31, 97.65) | 68.97 (51.06, 86.87) | 61.43 (54.79, 68.07) |
| Score ⩾20 | 14.85 (7.80, 21.91) | 95.65 (92.21, 99.10) | 71.43 (50.36, 92.50) | 60.55 (54.01, 67.09) |
| **PHQ-15 (Total)** | **SE (%) (95 %CI)** | **SP (%) (95 %CI)** | **NPV (%) (95 %CI)** | **PPV (%) (95 %CI)** |
| Score ⩾5 | 92.77 (89.43, 96.10) | 25.27 (21.28, 29.26) | 38.86 (34.81, 42.90) | 87.22 (81.47, 92.97) |
| Score ⩾6 | 89.36 (85.39, 93.33) | 32.68 (28.37, 36.99) | 40.46 (36.23, 44.70) | 85.71 (80.48, 90.95) |
| Score ⩾7 | 84.68 (80.04, 89.32) | 40.31 (35.80, 44.81) | 42.07 (37.61, 46.54) | 83.71 (78.80, 88.62) |
| Score ⩾8 | 79.57 (74.38, 84.77) | 52.07 (47.48, 56.66) | 45.95 (41.08, 50.81) | 83.28 (78.93, 87.62) |
| Score ⩾9 | 71.92 (66.13, 77.70) | 59.04 (54.53, 63.56) | 47.34 (42.13, 52.54) | 80.42 (76.16, 84.67) |
| Score ⩾10 | 63.83 (57.64, 70.02) | 65.58 (61.21, 69.94) | 48.70 (43.09, 54.31) | 77.98 (73.83, 82.13) |
| Score ⩾11 | 57.02 (50.65, 63.40) | 74.07 (70.05, 78.10) | 52.96 (46.77, 59.16) | 77.10 (73.16, 81.03) |
| Score ⩾12 | 49.36 (42.92, 55.80) | 79.74 (76.05, 83.43) | 55.50 (48.71, 62.30) | 75.46 (71.62, 79.31) |
| Score ⩾13 | 42.13 (35.77, 48.49) | 84.31 (80.97, 87.65) | 57.90 (50.42, 65.37) | 74.00 (70.22, 77.77) |
| Score ⩾14 | 35.32 (29.16, 41.47) | 87.80 (84.79, 90.80) | 59.71 (51.46, 67.97) | 72.61 (68.89, 76.33) |
| Score ⩾15 | 31.49 (25.51, 37.47) | 90.85 (88.20, 93.50) | 63.79 (54.92, 72.67) | 72.15 (68.48, 75.81) |
| Score ⩾16 | 26.81 (21.10, 32.51) | 93.25 (90.94, 95.55) | 67.02 (57.34, 76.70) | 71.33 (67.70, 74.96) |
| Score ⩾17 | 19.15 (14.08, 24.22) | 94.34 (92.21, 96.46) | 63.38 (51.90, 74.86) | 69.50 (65.88, 73.13) |
| Score ⩾18 | 15.75 (11.05, 20.44) | 96.08 (94.30, 97.86) | 67.27 (54.47, 80.07) | 69.01 (65.42, 72.61) |
| Score ⩾19 | 11.49 (7.38, 15.60) | 96.73 (95.10, 98.36) | 64.29 (49.17, 79.40) | 68.10 (64.51, 71.69) |
| Score ⩾20 | 8.94 (5.26, 12.61) | 97.82 (96.48, 99.16) | 67.74 (50.31, 85.17) | 67.72 (64.15, 71.29) |

PHQ-15: Patient Health Questionnaire15. Bio: Biomedical Settings. TCM: Traditional Chinese Medicine Settings. Psycho: Psychosomatic Settings. Total: Patients from all three settings. SE: Sensitivity. SP: Specificity. PPV: Positive Predictive Values. NPV: Negative Predictive Values. CI: confidence intervals.
